# Risk Factors and Prognosis for Metastatic Follicular Thyroid Cancer

**DOI:** 10.3389/fendo.2022.791826

**Published:** 2022-03-01

**Authors:** Ming-Hsien Wu, Yi-Yin Lee, Yu-Ling Lu, Shu-Fu Lin

**Affiliations:** ^1^ Division of Endocrinology and Metabolism, Department of Internal Medicine, New Taipei Municipal TuCheng Hospital (Built and Operated by Chang Gung Medical Foundation), New Taipei City, Taiwan; ^2^ Division of Endocrinology and Metabolism, Department of Internal Medicine, Chang Gung Memorial Hospital, Taoyuan, Taiwan; ^3^ College of Medicine, Chang Gung University, Taoyuan, Taiwan

**Keywords:** follicular thyroid cancer (FTC), distant metastasis, cancer-specific survival, risk factors, prognosis

## Abstract

**Background:**

Follicular thyroid cancer (FTC) is the second most common malignancy of thyroid. About 7%–23% of patients with FTC have distant metastasis. The aim of this study was to investigate the risk factors associated with distant metastasis and the impact of distant metastasis on survival in FTC patients.

**Methods:**

Patients with FTC were analyzed using a prospectively maintained dataset of thyroid cancer registered at a tertiary hospital in Taiwan between December 1976 and May 2020.

**Results:**

A total of 190 patients with a mean follow-up of 7.7 years were included in this study, including 29 with distant metastasis at diagnosis, 14 who developed metastasis during follow-up, and 147 without metastasis. Multivariate analysis adjusted for age, gender, tumor stage, and extrathyroidal invasion revealed old age (≥ 55 years) (adjusted odds ratio, 27.6; 95% confidence interval [CI], 8.75–86.8; P < 0.001) and extrathyroidal invasion (odds ratio, 24.1; 95% CI, 3.50–166.5; P = 0.001) were significantly associated with an increased risk of distant metastasis. Metastasis was correlated with higher cancer-specific mortality (adjusted hazard ratio, 35.5; 95% CI, 6.1–206.1; P < 0.001). In addition, patients with metastatic FTC diagnosed on initial presentation had the lowest 10-year cancer-specific survival rate (26.0%), followed by those who developed metastatic disease after initial treatment (76.6%), while patients without metastasis were all alive (100%) (P ≤ 0.002 for all comparisons).

**Conclusions:**

Age and extrathyroidal invasion are significant risk factors for distant metastasis of FTC. Patients with metastatic FTC, especially when diagnosed on initial presentation, have dismal survival outcomes.

## Introduction

Thyroid cancer is the most common endocrine malignancy, with a rising incidence worldwide over the past four decades (1, 2). Follicular thyroid cancer (FTC) and papillary thyroid cancer (PTC), collectively referred to as differentiated thyroid cancer, account for the majority (>85%) of thyroid cancer (3). However, FTC differs markedly from PTC, including in pathological findings, genetic alterations, and worse prognosis (3–7).

FTC is more likely to metastasize to distant organs, including the lungs and bone, rather than regional cervical lymph node metastasis (4). Distant metastasis is observed in up to 7%–23% of FTC patients (8, 9). Metastasis is identified as the most significant prognostic factor affecting survival in FTC (6, 10). Distant metastasis can be an initial presentation of FTC, and it may develop after initial treatment. A study evaluated the outcomes of FTC patients who had distant metastasis at presentation versus those who developed metastatic disease during the follow-up period (8). The results demonstrated a similar risk of mortality between these two groups. However, this finding should be interpreted with caution due to the limited number of patients in the early metastasis group (n = 12) and delayed metastasis group (n = 8). Additional studies with larger sample sizes are needed to clarify the significance of the timing of metastasis on mortality in FTC patients (11).

In this study, we evaluated the risk factors associated with distant metastasis in patients with FTC, and determined the impact of metastasis, both diagnosed at presentation and developed during follow-up, on cancer-specific survival (CSS).

## Materials and Methods

### Source of Data

This study investigated data in a thyroid cancer dataset at Chang Gung Memorial Hospital in Taiwan. This dataset was created to facilitate research on thyroid cancer (12, 13). Information for all patients with thyroid cancer treated at this medical center was prospectively collected. Clinical features, imaging results, laboratory data, pathological findings, treatment, and follow-up were extensively recorded.

### Study Cohort

A total of 460 FTC patients were identified in the dataset between December 1976 and May 2020. Patients were excluded if they met the following exclusion criteria: received the primary surgery at other hospitals, lost to follow-up, had insufficient medical data, and local recurrent disease. Systematic validation of these data using the electronic health record was performed before further analyses. Eligible patients were divided into three groups depending on the clinical course of distant metastasis: distant metastasis detected at presentation (M1), presence of distant metastasis during follow-up (M2), and no evidence of distant metastasis during the study period (M0).

### Covariates and Study Outcomes

Covariates that could potentially affect distant metastasis and survival, including patient age, gender, TNM stage, extrathyroidal invasion, primary surgery, radioiodine therapy, external beam radiation therapy (EBRT), and targeted therapy were identified and included in our analyses (14–17). The 8^th^ edition American Joint Committee on Cancer (AJCC) staging system for FTC was implemented in this study (18).

### Ethics Statement

The study was approved by the Ethics Committee on Research of the Institutional Review Board at Chang Gung Memorial Hospital (No. 202001231B0), and was conducted in accordance with the principles of the Helsinki Declaration. Informed consent was waived by the Institutional Review Board at Chang Gung Memorial Hospital.

### Statistical Analysis

Continuous variables with normal distribution were presented as the mean ± standard deviation. Continuous variables without normal distribution were presented as the median and interquartile range (IQR). Categorical variables were presented as number and percentage. The comparisons of the characteristics were calculated using an independent samples *t*-test or one-way analysis of variance (ANOVA) for continuous variables with normal distribution. The Mann-Whitney U test and Kruskal-Wallis ANOVA were used for continuous variables without normal distribution, and the chi-squared test was used for categorical variables.

Binary logistic regression was used to determine which variables were associated with distant metastasis. Cox regression analysis was performed to determine the factors associated with cancer-specific mortality. Kaplan–Meier survival curves were applied to estimate the CSS. The log-rank test was used to determine the 10-year and 20-year CSS rates. The analysis was performed using SPSS statistical software (version 22.0, SPSS Inc., Chicago, IL); P < 0.05 was considered statistically significant.

## Results

### Demographic and Clinical Features

**Figure 1:**
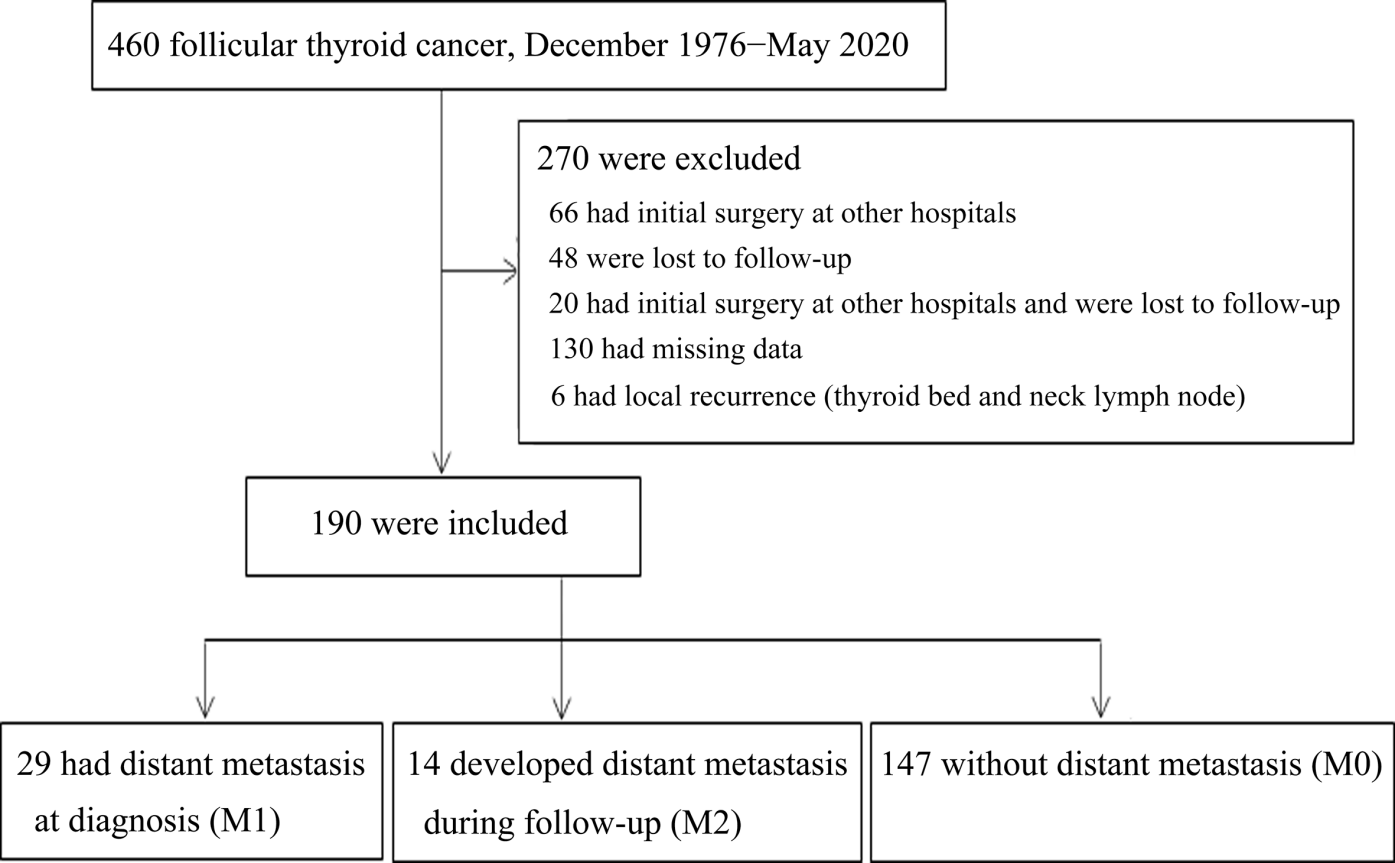
Flowchart of cohort establishment.

**Figure 1** presents the flowchart for the identification of three cohorts of FTC patients according to the status of distant metastasis, including metastasis identified at presentation (M1), metastasis identified during the follow-up period (M2), and no evidence of metastasis (M0). A total of 460 patients with FTC were identified in our thyroid cancer registry dataset at Chang Gung Memorial Hospital in Taiwan between December 1976 and May 2020. Patients were excluded if they met the exclusion criteria, including 66 patients who had initial surgery at other hospitals, 48 who were lost to follow-up, 20 who had initial surgery at other hospitals and also lost to follow-up, 130 who had missing data identified during the validation process using electronic health record, and 6 with local recurrence (thyroid bed and neck lymph node) and without metastasis. Therefore, 190 patients with FTC diagnosed between August 1987 and July 2018 were included for further analysis, including 29 in the M1 group, 14 in the M2 group, and 147 in the M0 group.

**Table 1** summarizes the demographic and clinical features of these 190 patients. The mean age at diagnosis of FTC in M1 group was the oldest (66.1 ± 8.9 years), followed by M2 group (59.7 ± 8.7 years), and M0 group (42.7 ± 17.3 years) (P < 0.001). The distribution of gender among the three groups was similar (P = 0.197). FTC tumors larger than 4 cm occurred in 34.5% patients in M1 group, 50.0% in M2 group, and 27.9% in M0 group (P = 0.027). Greater percentages of patients with an advanced T stage (T3/T4) appeared in M1 group (34.4%) and M2 group (71.4%), and fewer in M0 group (27.9%) (P < 0.001). The percentages of lymph node metastasis were lower in M1 group (3.4%), comparing to M2 group (14.3%) and M0 group (11.6%). However, the differences of lymph node metastasis did not reach statistical significance among these three cohorts (P = 0.479) or between groups (P > 0.05). The M1 group had the highest proportion of patients with an advanced TNM stage (stage II/III/IV) (100%), followed by M2 group (57.1%) and M0 group (11.6%) (P < 0.001). The proportions of extrathyroid invasion were higher in M1 group (17.2%%) and M2 group (35.7%), comparing to M0 group (2.0%) (P < 0.001). The most common site of metastasis was bone (51.7%) in M1 group, while lung was the most common metastatic organ (85.7%) in M2 group (P < 0.001). The extent of thyroid surgery among the three groups was similar (P = 0.869). Greater percentages of patients underwent radioiodine therapy in M1 group (100%) and M2 group (100%), and fewer in M0 group (81.6%) (P < 0.001). The median cumulative radioiodine dose was higher in M1 group (490.0 mCi), followed by M2 group (380.0 mCi) and M0 group (30.0 mCi) (P < 0.001). EBRT and targeted therapy (sorafenib and lenvatinib) were used in some patients in M1 group (20.7% and 10.3%, respectively) and M2 group (28.6% and 7.1%, respectively), while no patients in M0 group received EBRT or targeted therapy. Patients in M1 group had the worst cancer-specific mortality (58.6%), followed by M2 group (28.6%), and no cancer-specific mortality occurred in M0 group (P < 0.001). The mean follow-up time in M1, M2 and M0 groups was 5.4 ± 4.3 years, 13.8 ± 8.6 years, and 7.6 ± 5.3 years, respectively.

**Table 1:** Clinical features of 190 patients with follicular thyroid cancer.

	M1 group, n=29	M2 group, n=14	M0 group, n=147	P value
Age (years)	66.1 ± 8.9	59.7 ± 8.7	42.7 ± 17.3	<0.001
Gender, n (%)				0.197
Female	20 (69.0%)	10 (71.4%)	121 (82.3%)	
Male	9 (31.0%)	4 (28.6%)	26 (17.7 %)	
Tumor size, n (%)				0.027
≤2 cm	11 (37.9%)	1 (7.1%)	28 (19.0%)	
>2-4 cm	8 (27.6%)	6 (42.9%)	78 (53.1%)	
>4 cm	10 (34.5%)	7 (50.0%)	41 (27.9%)	
T stage, n (%)				<0.001
T1	11 (37.9%)	0 (0%)	28 (19.0%)	
T2	8 (27.6%)	4 (28.6%)	78 (53.1%)	
T3	7 (24.1%)	6 (42.8%)	41 (27.9%)	
T4	3 (10.3%)	4 (28.6%)	0 (0%)	
N stage, n (%)				0.479
N0	28 (96.6%)	12 (85.7%)	130 (88.4%)	
N1	1 (3.4%)	2 (14.3%)	17 (11.6%)	
M stage, n (%)				<0.001
M0	0 (0%)	14 (100.0%)	147 (100%)	
M1	29 (100.0%)	0 (0%)	0 (0%)	
TNM stage at diagnosis, n (%)				<0.001
Stage I	0 (0%)	6 (42.9%)	130 (88.4%)	
Stage II	2 (6.9%)	6 (42.9%)	17 (11.6%)	
Stage III	0 (0%)	1 (7.1%)	0 (0%)	
Stage IV	27 (93.1%)	1 (7.1%)	0 (0%)	<0.001
Extrathyroidal invasion, n (%)	5 (17.2%)	5 (35.7%)	3 (2.0%)	
Initial site of metastasis, n (%)				<0.001
Lung	11* (37.9%)	12 (85.7%)	0 (0%)	
Bone	15* (51.7%)	2 (14.3%)	0 (0%)	
Skin	3 (10.3%)	0 (0%)	0 (0%)	
Gum	1 (3.4%)	0 (0%)	0 (0%)	
Thyroid surgery, n (%)				0.869
Lobectomy or subtotal thyroidectomy	6 (20.7%)	3 (21.4%)	37 (25.2%)	
Total thyroidectomy	23 (79.3%)	11 (78.6%)	110 (74.8%)	
^131^I therapy, n (%)	29 (100.0%)	14 (100.0%)	120 (81.6%)	<0.001
^131^I median cumulative dose (mCi, IQR)	490.0 (30, 760)	380.0 (63.8, 742.5)	30.0 (30.0, 90.0)	<0.001
External beam radiation therapy, n (%)	6 (20.7%)	4 (28.6%)	0 (0%)	<0.001
Targeted therapy, n (%)^#^	3 (10.3%)	1 (7.1%)	0 (0%)	0.002
Cancer-specific mortality, n (%)	17 (58.6%)	4 (28.6%)	0 (0%)	<0.001
Follow-up (years)	5.4 ± 4.3	13.8 ± 8.6	7.6 ± 5.3	<0.001

*One patient had lung and bone metastasis simultaneously at diagnosis.

^#^Including sorafenib and lenvatinib.

### Risk Factor for Distant Metastasis

Among 190 FTC patients, 29 (15.3%) had distant metastasis at diagnosis and 14 (7.4%) developed metastasis during follow-up. Risk factors for metastasis were analyzed (**Table 2**). The univariate logistic regression demonstrated that metastasis was significantly associated with old age (≥55 years) (odds ratio, 22.6; 95% confidence interval [CI], 8.28–61.7; P < 0.001), advanced T stage (T3/T4) (odds ratio, 2.25; 95% CI, 1.12–4.52; P = 0.023) and extrathyroidal invasion (odds ratio, 14.5; 95% CI, 3.79–55.8; P < 0.001). Multivariate logistic regression adjusted for age, gender, T stage, N stage and extrathyroidal invasion indicated that old age (adjusted odds ratio, 27.6; 95% CI, 8.75–86.8; P < 0.001) and extrathyroidal invasion (adjusted odds ratio, 24.1; 95% CI, 3.50–166.5; P = 0.001) were significantly correlated with increased risk of distant metastasis.

**Table 2:** Risk factors associated with distant metastasis in 190 patients with follicular thyroid cancer.

Variable	Univariate OR (95% CI)	P value	Multivariate OR (95% CI)^a^	P value
Age (years) (≥ 55 vs < 55)	22.6 (8.28-61.7)	<0.001	27.6 (8.75-86.8)	<0.001
Gender (male vs female)	0.50 (0.23-1.08)	0.077	0.34 (0.12-0.97)	0.044
T stage (T3 and T4 vs T1 and T2)	2.25 (1.12-4.52)	0.023	0.82 (0.31-2.17)	0.695
N stage (N1 vs N0)	0.57 (0.16-2.06)	0.394	0.75 (0.16-3.58)	0.722
Extrathyroidal invasion (yes vs no)	14.5 (3.79-55.8)	<0.001	24.1 (3.50-166.5)	0.001

a Adjusted for age, gender, T stage, N stages and extrathyroidal invasion.

### Risk Factors for Cancer-Specific Mortality

We sought to identify risk factors for cancer-specific mortality in these 190 FTC patients (**Table 3**). Univariate Cox regression analysis revealed that cancer-specific mortality was significantly correlated with old age (≥55 years; hazard ratio, 34.1; 95% CI, 4.57–255.1; P = 0.001), metastasis (hazard ratio, 36.2; 95% CI, 12.01–109.0; P < 0.001), advanced TNM stage (stage II/III/IV; hazard ratio, 402.1; 95% CI, 2.9–55.8 x 10^3^; P = 0.017), extrathyroidal invasion (hazard ratio, 3.00; 95% CI, 1.06–8.51; P = 0.039), ^131^I cumulative dose ≥100 mCi (hazard ratio, 11.5; 95% CI, 2.61–50.2; P = 0.001), and EBRT (hazard ratio, 10.0; 95% CI, 4.1–24.6; P < 0.001). In a multivariate Cox regression analysis adjusted for age, gender, T stage, N stage, M stage, extrathyroidal invasion, primary surgery, ^131^I cumulative dose, EBRT, and targeted therapy, only distant metastasis was significantly associated with increased risk of cancer-specific mortality (adjusted hazard ratio, 35.5; 95% CI, 6.1–206.1; P < 0.001).

**Table 3:** Risk factors associated with cancer-specific mortality in 190 patients with follicular thyroid cancer.

	Univariate	P value	Multivariate	P value
	HR (95% CI)		Adjusted HR (95% CI)^a^	
Age (years)				
<55	Reference		Reference	
≥55	34.1 (4.57-255.1)	0.001	5.85 (0.51-66.6)	0.155
Gender				
Male	Reference			
Female	1.01 (0.36-2.81)	0.988		
T stage				
T1 and T2	Reference			
T3 and T4	1.56 (0.66-3.70)	0.312		
N stage				
N0	Reference			
N1	0.22 (0.03-1.70)	0.148		
M stage				
M0	Reference		Reference	
M1	36.2 (12.01-109.0)	<0.001	35.5 (6.1-206.1)	<0.001
TNM stage				
I	Reference		–	–
II, III, IV	402.1 (2.9-55.8 × 10^3^)	0.017	–	–
Extrathyroidal invasion				
No	Reference		Reference	
Yes	3.00 (1.06-8.51)	0.039	0.66 (0.15-2.80)	0.569
Primary surgery				
Total thyroidectomy	Reference			
Less than total thyroidectomy	0.86 (0.29-2.59)	0.863		
^131^I cumulative dose (mCi)				
<100	Reference		Reference	
≥100	11.5 (2.61-50.2)	0.001	0.51 (0.09-3.01)	0.455
External beam radiation therapy				
No	Reference		Reference	
Yes	10.0 (4.1-24.6)	<0.001	0.69 (0.21-2.30)	0.544
Targeted therapy^#^				
No	Reference			
Yes	20.5 (0.00-2.2 × 10^6^)	0.776		

a Adjusted for age, gender, T stage, N stage, M stage, extrathyroidal invasion, primary surgery, ^131^I cumulative dose, external beam radiation therapy, and targeted therapy.

^#^Including sorafenib and lenvatinib.

### Cancer-Specific Survival

**Figure 2:**
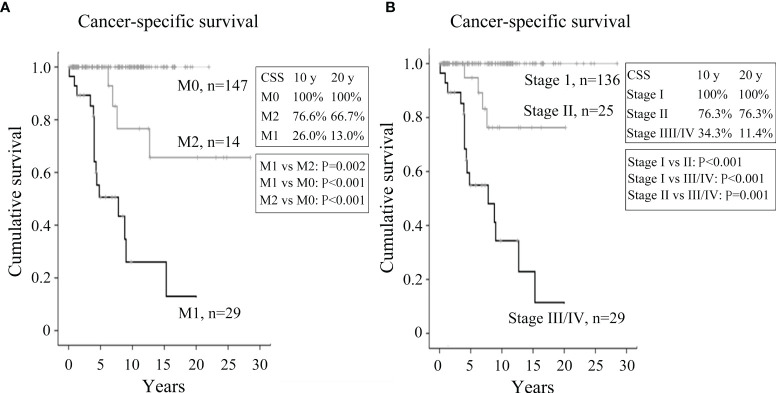
Kaplan–Meier plots illustrating cancer-specific survival of FTC patients. **(A)** Kaplan–Meier survival plots for patients with metastatic FTC at presentation (M1), developing metastasis during follow-up (M2), and with no evidence of metastasis (M0). **(B)** Kaplan–Meier survival plots for FTC patients with stage I, II, and III/IV disease at diagnosis.

CSS was evaluated using the Kaplan–Meier method with the log-rank test. CSS was significantly different between the three cohorts (**Figure 2A**). The M1 group had the worst CSS with 10-year and 20-year CSS rates of 26.0% and 13.0%, respectively, followed by the M2 group (76.6% and 66.7%) and M0 group (100% and 100%) (P ≤ 0.002 for all comparisons). We also analyzed the CSS according to the 8^th^ edition of TNM staging system of the AJCC (**Figure 2B**) (18). As expected, patients with stage III/IV disease had the worst CSS, with 10-year and 20-year survival rates of 34.3% and 11.4%, respectively, followed by those with stage II disease (76.3% and 76.3%) and stage I disease (100% and 100%) (P ≤ 0.001 for all comparisons). Patients with stage III and IV disease were analyzed collectively in this study due to the limited number of patients with stage III disease (n = 1).

### Demographic and Clinical Features of 14 Patients Developed Metastasis During Follow-up

A total of 14 patients developed distant metastasis during follow-up (**Table 4**). The median time from FTC diagnosis to distant metastasis was 9.6 years (0.8–19.5 years). There were four male and ten female patients aged between 38 and 70 years. The median size of FTC was 4.0 cm (2.0–18.0 cm). The initial TNM stages were categorized into I (42.9%), II (42.9%), III (7.1%), and IV (7.1%). There were 4 patients with extrathyroidal invasion of tumor during operation. Most patients (78.6%) received a total thyroidectomy at diagnosis of FTC, and all (100%) underwent radioiodine treatment with a median cumulative dose of 380 mCi (30–1215 mCi). The primary sites of metastasis were the lung (85.8%), followed by bone (14.2%). Three patients (21.4%) developed lung and bone metastases sequentially. Of the 12 patients with lung metastasis, 3 (25%) underwent surgery for lung lesions. Of the 5 patients with bone metastasis, 4 (80%) received palliative EBRT for bone lesions. One patient received targeted therapy (lenvatinib). Four patients died of FTC, including those 3 patients developing both lung and bone metastases.

**Table 4:** Clinical features of 14 patients who developed metastatic disease during follow-up.

Patient	Age/Gender	Tumor size (cm)	Stage	Extrathyroidal invasion	Operation method	^131^I cumulative dose (mCi)	First site of metastasis	Second site of metastasis	Operation for lung metastasis	Radiation therapy for bone metastasis	Targeted therapy	Interval between initial diagnosis and first distant metastasis (years)	Follow-up period (years)	Cancer-specific mortality
1	68/F	8.0	T3N0M0, stage II	No	Total thyroidectomy	830	Lung	Bone	Yes	Yes	No	3.7	7.6	Yes
2	65/F	3.5	T3N0M0, stage II	No	Lobectomy	780	Lung	Bone	No	Yes	No	2.3	6.9	Yes
3	59/F	18.0	T4N0M0, stage IV	Yes	Lobectomy	1215	Lung	Bone	No	Yes	No	12	12.7	Yes
4	70/F	5.0	T3N0M0, stage II	No	Total thyroidectomy	30	Lung	No	No	No	No	3.6	6.2	Yes
5	53/M	5.0	T3N0M0, stage I	No	Total thyroidectomy	235	Lung	No	Yes	No	No	19.5	23.1	No
6	55/F	2.8	T2N0M0, stage I	No	Total thyroidectomy	175	Lung	No	No	No	No	14.4	24.8	No
7	49/F	5.0	T4N0M0, stage I	Yes	Total thyroidectomy	360	Lung	No	No	No	No	13.6	28.5	No
8	58/M	4.4	T3N0M0, stage II	No	Subtotal thyroidectomy	400	Lung	No	Yes	No	No	13.0	20.2	No
9	63/F	3.6	T2N0M0, stage I	No	Total thyroidectomy	350	Lung	No	No	No	No	9.0	11.1	No
10	38/M	2.0	T4N1M0, stage I	Yes	Total thyroidectomy	730	Lung	No	No	No	No	11.9	24.3	No
11	65/F	3.9	T4N0M0, stage III	Yes	Total thyroidectomy	700	Lung	No	No	No	No	2.0	12.5	No
12	64/M	4.0	T2N0M0, stage I	No	Total thyroidectomy	30	Lung	No	Yes	No	Lenvatinib	0.8	1.8	No
13	67/F	5.7	T3N0M0, stage II	No	Total thyroidectomy	450	Bone	No	No	Yes	No	3.6	6.3	No
14	62/F	2.4	T2N1M0, stage II	No	Total thyroidectomy	130	Bone	No	No	No	No	10.2	7.2	No

### Risk Factors for Mortality in 14 Patients Developing Metastasis During Follow-Up

Risk factors for cancer-specific mortality were analyzed for M2 group (**Table 5**). The data revealed that multiple sites of metastasis and EBRT were significantly associated with cancer-specific mortality (P = 0.011 and P = 0.041, respectively), while age, gender, tumor size, T stage, N stage, TNM stage, type of thyroid surgery, ^131^I cumulative dose, time from FTC diagnosis to distant metastasis, and targeted therapy did not reveal any association. The number of patients was too small to conduct multivariate analysis.

**Table 5:** Risk factors associated with mortality in 14 patients who developed metastasis during follow-up.

	Survival group, n=10	Mortality group, n=4	*p* value
Age (years)	57.4 ± 8.9	65.5 ± 4.8	0.117
Gender, n (%)			0.251
Female	6 (60)	4 (100)	
Male	4 (40)	0 (0)	
Tumor size, n (%)			0.685
≤2 cm	1 (10)	0 (0)	
>2-4 cm	5 (50)	1 (25)	
>4 cm	4 (40)	3 (75)	
T stage, n (%)			0.293
T1	0 (0)	0 (0)	
T2	4 (40)	0 (0)	
T3	3 (30)	3 (75)	
T4	3 (30)	1 (25)	
N stage, n (%)			0.560
N0	8 (80)	4 (100)	
N1	2 (29)	0 (0)	
TNM stage, n (%)			0.140
I	6 (60)	0 (0)	
II	3 (30)	3 (75)	
III	1 (10)	0 (0)	
IV	0 (0)	1 (25)	
Extrathyroidal invasion, n (%)	3 (30)	1 (25)	0.597
Thyroid surgery, n (%)			0.176
Lobectomy or subtotal thyroidectomy	1 (10)	2 (50)	
Total thyroidectomy	9 (90)	2 (50)	
^131^I cumulative dose (mCi)	356.0 ± 229.4	713.8 ± 495.5	0.081
Interval between initial diagnosis and first metastasis (years)	9.8 ± 6.0	5.4 ± 4.5	0.214
Multiple metastatic sites, n (%)	0 (0)	3 (75)	0.011
Radiation therapy, n (%)	1 (10)	3 (75)	0.041
Targeted therapy, n (%)	1 (10)	0 (0)	1.000

## Discussion

By analyzing a prospectively maintained dataset, we found that FTC patients without metastatic disease had a favorable prognosis with a 10-year CSS rate of 100%. However, the outcome for patients with metastatic disease, diagnosed either at presentation or during follow-up, was poor, with 10-year CSS rates of 26.0% and 76.6%, respectively. Our data are similar to a study that revealed CSS rates at 10 years were 41% and 76% for patients with metastatic FTC diagnosed at presentation and during follow-up, respectively (19). We additionally demonstrated that FTC patients with metastasis at diagnosis had worse outcomes than those who developed metastasis during follow-up.

Prior reports have demonstrated old age, large tumor size, aggressive histological classification, vascular invasion, extrathyroidal invasion, and metastasis were predictors for cancer-specific mortality in FTC patients (10, 14–16, 20, 21). In this study, we found metastasis was an independent risk factor for cancer-specific mortality, while old age, gender, T stage, N stage, extrathyroid extension, and method of primary surgery were not. Our data indicate distant metastasis is the most important prognostic factor for FTC mortality.

In our study, 22.6% (43/190) of FTC patients had metastatic disease at diagnosis or during follow-up. The risk factors associated with distant metastasis were old age and extrathyroidal invasion. This finding is consistent with a prior report revealing old age was a significant risk factor to develop metastatic FTC (19).

Bone was the main site of metastasis (51.7%) in M1 group. In contrast, the lungs were the most common initial distant metastasis site (85.7%) in M2 group. The underlying mechanisms accounting for the difference in initial metastatic organs between M1 group and M2 group are unclear and need to be clarified. Patients with bone metastasis usually have poorer outcomes than those with lung metastasis (22, 23). The potential explanations for the worse outcomes in patients with bone metastasis may be related to immobilization arising from bone metastasis, complications associated with bone metastasis (hypercalcemia, pathological bone fractures, and spinal cord compression), and no effective treatments for bone metastasis (17, 24). We found that patients with multiple organs with metastasis (bone and lung) had increased mortality risks compared with those having single-organ metastasis.

A prior study reveals old age is a risk factor for cancer mortality in patients with FTC (25). However, our data of 14 patients who developed metastasis during follow-up failed to support that old age is associated with increased risk of FTC mortality. One of the potential explanations could be the limited patient number in survival group (n = 10) and mortality group (n = 4). Of note, all patients (n = 3) below 55 years had long-term survival (> 23.1−28.5 years).

Ten patients received EBRT in this study. EBRT is typically reserved for control of unresectable locoregional FTC, metastatic bone disease for pain relief and spinal cord compression (24).

Sorafenib and lenvatinib are two tyrosine kinase inhibitors approved for patients with progressive radioiodine-refractory FTC (3, 26, 27). These drugs were used in 4 patients in this study. Previous data revealed progression disease developed 10.8 and 18.3 months after the initiation of sorafenib and lenvatinib treatment, respectively (25, 26). Sorafenib and lenvatinib frequently induce adverse events that result in dose reduction or treatment termination. Novel therapies with different therapeutic mechanisms are mandatory to improve the outcomes in patients with refractory FTC (28).

This study has some limitations. First, 270 patients with FTC were excluded in this study due to meet the exclusion criteria. However, the number of FTC patients included in our analyses was larger than that of prior studies (8, 19), including the total number of patients with FTC (190 vs. 91–134), and the number of patients with metastatic FTC (43 vs. 20–36). Second, we did not include histological subtypes of FTC for analysis. The WHO 2017 classification for FTC includes minimally invasive FTC, encapsulated angioinvasive FTC, and widely invasive FTC (29). Widely invasive FTC usually appears in older patients, with larger tumor sizes, invasive growth into the thyroid, and distant metastasis (30). Minimally invasive FTC has excellent prognosis, while encapsulated angioinvasive FTC and widely invasive FTC have higher risk of metastasis and recurrence. Hemithyroidectomy is recommended for minimally invasive FTC. Total thyroidectomy with postoperative radioiodine therapy is required for angioinvasive FTC and widely invasive FTC. Prophylactic central lymph node dissection is not necessary due to the risk of postoperative complications and a lack of therapeutic benefit (31, 32). Most of our patients received total thyroidectomy (75.8%) and radioiodine therapy (85.8%). Third, this dataset lacks genetic mutation profile information, such as *TERTp* and *EIF1AX*, which may help predict a worse outcome in these patients (33). Additional studies are needed to address this issue.

## Conclusions

Age is a significant risk factor for distant metastasis of FTC. FTC patients without metastatic disease have promising prognosis. However, metastatic FTC, either diagnosed at presentation or during follow-up, have poor survival outcomes.

## Data Availability Statement

The original contributions presented in the study are included in the article/supplementary material. Further inquiries can be directed to the corresponding author.

## Ethics Statement

This study was approved by Institutional Review Board of Chang Gung Medical Foundation (No. 202001231B0).

## Author Contributions

M-HW and S‐FL conceived the study. M-HW and S‐FL designed the study. M-HW, Y-YL, and Y-LL acquired and analyzed the data. All authors interpreted the data. M-HW, and S‐FL wrote the manuscript. All authors reviewed and revised the manuscript.

## Funding

This study was supported by the New Taipei Municipal TuCheng Hospital (built and operated by Chang Gung Medical Foundation), New Taipei City, Taiwan (CMRPVVJ0012), and Chang Gung Memorial Hospital, Linkou, Taiwan (CMRPG3J0491).

## Conflict of Interest

The authors declare that the research was conducted in the absence of any commercial or financial relationships that could be construed as a potential conflict of interest.

## Publisher’s Note

All claims expressed in this article are solely those of the authors and do not necessarily represent those of their affiliated organizations, or those of the publisher, the editors and the reviewers. Any product that may be evaluated in this article, or claim that may be made by its manufacturer, is not guaranteed or endorsed by the publisher.
